# Membrane Stabilization and Detoxification of Acetaminophen-Mediated Oxidative Onslaughts in the Kidneys of Wistar Rats by Standardized Fraction of* Zea mays* L. (Poaceae),* Stigma maydis*

**DOI:** 10.1155/2016/2046298

**Published:** 2016-08-07

**Authors:** S. Sabiu, F. H. O'Neill, A. O. T. Ashafa

**Affiliations:** ^1^Phytomedicine and Phytopharmacology Research Group, Department of Plant Sciences, University of the Free State, QwaQwa Campus, Phuthaditjhaba 9866, South Africa; ^2^Department of Microbial, Biochemical, and Food Biotechnology, University of the Free State, Nelson Mandela Drive, P.O. Box 339, Bloemfontein 9301, South Africa

## Abstract

This study evaluated membrane stabilization and detoxification potential of ethyl acetate fraction of* Zea mays *L.,* Stigma maydis* in acetaminophen-induced oxidative onslaughts in the kidneys of Wistar rats. Nephrotoxic rats were orally pre- and posttreated with the fraction and vitamin C for 14 days. Kidney function, antioxidative and histological analyses were thereafter evaluated. The acetaminophen-mediated significant elevations in the serum concentrations of creatinine, urea, uric acid, sodium, potassium, and tissue levels of oxidized glutathione, protein-oxidized products, lipid peroxidized products, and fragmented DNA were dose-dependently assuaged in the fraction-treated animals. The fraction also markedly improved creatinine clearance rate, glutathione, and calcium concentrations as well as activities of superoxide dismutase, catalase, glutathione reductase, and glutathione peroxidase in the nephrotoxic rats. These improvements may be attributed to the antioxidative and membrane stabilization activities of the fraction. The observed effects compared favorably with that of vitamin C and are informative of the fraction's ability to prevent progression of renal pathological conditions and preserve kidney functions as evidently supported by the histological analysis. Although the effects were prominently exhibited in the fraction-pretreated groups, the overall data from the present findings suggest that the fraction could prevent or extenuate acetaminophen-mediated oxidative renal damage via fortification of antioxidant defense mechanisms.

## 1. Introduction 

The kidney is a highly specialized organ that maintains the body's homeostasis by selectively excreting or retaining various substances according to specific body needs. In its role as a detoxifier and primary eliminator of xenobiotics, it becomes vulnerable to developing injuries. Such injuries have been linked with reactive oxygen species (ROS) mediated oxidative stress on renal biomolecules [1]. The kidney's response to toxicants varies by multiple morphological patterns beginning with tubular or interstitial changes to nephropathy [2]. Kidney disorders account for 1 in 10 deaths, making Chronic Kidney Disease (CKD) one of the most sought after public health concerns in recent years [3]. The prevalence of the disease is more disconcerting in sub-Saharan Africa countries like Nigeria and South Africa with an estimation of 23 and 40%, respectively [3, 4]. Till date, orthodox management therapies for kidney disorders have been embraced and identified to include the use of renal replacement therapy (dialysis and transplantation) and applications of angiotensin-converting enzyme (ACE) inhibitors, angiotensin II receptor blockers (ARBs), and erythropoietin to slow the progression of loss of kidney function [5]. The affordability, sensitivity, and inherent adverse effects of the aforementioned therapies have undermined their applications in the past. The availability of kidneys for transplantation and cost are other important challenges consistent with renal replacement therapy [6]. Interestingly, traditional systems of medicine have offered effective drugs against kidney pathological conditions and thus can be used to protect renal function and prevent/slow the progression of renal diseases to CKD or end stage renal disease [7]. A number of drugs from herbal sources have been shown to be nephroprotective and there is a keen global interest on the development of such. The focus is mostly to protect or prevent injurious insults to the kidney as well as enhance the regeneration of tubular cells [8].

*Zea mays *L. (Poaceae),* Stigma maydis* (corn silk) is one of several herbs commonly used in the management of kidney stones, bedwetting, and urinary infections [9]. GCMS analysis of its aqueous extract from our laboratory revealed the presence of maizenic acid, *β*-carotene, ascorbic acid, gluten, o-diethyl phthalate, 2-methyl-naphthalene, thymol, 3′-o-methyl-maysin, cyanidin, cinnamic acid, hordenine, luteolinidin, pelargonidin, and betaine as major identifiable adaptogenic phytonutrients [10]. Corn silk (CS) has found therapeutic applications as an insecticide, disinfectant, antioxidant, antibiotic, and immune booster [11–13]. Its pharmacological significance in Asian folkloric medicine as oral hypoglycemic and anti-inflammatory agents has also been reported [14–16]. Lamentably in Africa, particularly, Nigeria and South Africa, where corn is a common staple food, CS is still underutilized and its pharmacological significance is chiefly untapped.

Besides very limited research on the therapeutic importance of CS in Africa, opinions on its nephroprotective potential are divergent. While Sukandar et al. [17] demonstrated the potency of its ethanolic extract against gentamicin/piroxicam-induced kidney failure, Sepehri et al. [18] submitted that treatment with its methanolic extract does not result in complete reversal of gentamicin-induced alterations in kidney function parameters. More comprehensive research is however imperative in this direction and has prompted the present study with a view to providing detailed biochemical information on the ability of CS to preserve renal functions and delay/prevent the progression of renal pathological conditions. Hence, we evaluated its standardized fraction against acetaminophen-perturbed oxidative onslaughts in the kidneys of Wistar rats. In addition, the membrane stabilization capacity of the CS fraction was also investigated.

## 2. Materials and Methods

### 2.1. Chemicals, Reagents, and Assay Kits

Assay kits for kidney function parameters, glutathione peroxidase, and glutathione reductase were purchased from Randox Laboratories Limited, United Kingdom. Acetaminophen (APAP) and vitamin C were products of Emzor Pharmaceuticals, Lagos, Nigeria. The water used was glass-distilled and all other chemicals and reagents were of analytical grade.

### 2.2. Plant Collection and Authentication

Fresh corn silks were harvested from a maize plantation in the Phuthaditjhaba area of Maluti-A-Phofung, QwaQwa, Free State province, South Africa, between November 2014 and March 2015. They were authenticated by Dr. A. O. T. Ashafa of the Plant Sciences Department, University of the Free State, QwaQwa Campus, South Africa. Voucher specimen (number SabMed/01/2015/QHB) was thereafter prepared and deposited at the Herbarium of the University.

### 2.3. Extract Processing, Standardization, and Selection

The CS was shade dried to constant weight and subsequently milled by an electric blender (model MS-223; Labcon PTY, Durban, South Africa) to fine powder. The powdered sample (2 kg) was extracted with 70% methanol (10 L) with regular agitation for 24 h. The solution obtained was filtered (Whatman no. 1 filter paper) and the resulting filtrate concentrated to a yield of 455 g crude extract. Part of the crude extract (400 g) was suspended in distilled water (0.6 L) and subsequently partitioned in succession with n-hexane, dichloromethane, ethyl acetate, and n-butanol. This yielded 18 g, 24 g, 33 g, and 42 g of the respective fractions. About 10 *μ*L of each (1 mg/mL) was spotted on silica gel TLC plates. The resulting chromatograms were thereafter developed in dichloromethane/methanol (8.5 : 1.5 v/v) solvent system and sprayed with 0.2% 1,1-diphenyl-2-picrylhydrazyl (DPPH) in methanol for detection of antioxidant metabolites. From the chromatograms, the ethyl acetate fraction of CS (CSEAF) had the highest number of antioxidant spots and was selected for the subsequent biochemical assays. CSEAF was kept air-tight and refrigerated prior to commencement of the study.

### 2.4. Experimental Animals

This study was approved (UFS-AED2015/0005) by the Ethical Committee of University of the Free State, South Africa, in accordance with the Guidelines of the National Research Council Guide for the Care and Use of Laboratory Animals [19] and principles of Good Laboratory Procedure [20]. Healthy Wistar rats (both sexes) of weight range 200–222 g were collected from the experimental animal facility of University of the Free State, Bloemfontein, South Africa. They were housed in clean metabolic cages placed in a well-ventilated animal house with optimal conditions (temperature 23 ± 1°C, photoperiod; 12 h natural light and 12 h dark; humidity; 45–50%). They were acclimatized to the animal house condition for 10 days and had* ad libitum* access to pelleted rat chow (Pioneer Food (Pty) Ltd., Huguenot, South Africa) and water.

### 2.5. Nephroprotective Study

#### 2.5.1. Induction of Renal Injury

This was achieved as previously described [21]. Briefly, the animals were fasted overnight for 14 h and a single oral dose of APAP (750 mg/kg body weight (b.w.)) was thereafter administered. These animals essentially represent nephrotoxic rats.

### 2.6. Experimental Protocol

Fifty rats randomized into 9 experimental groups were used for this study. While the nephrotoxic control group had 10 animals that were further divided into 2 sets (with one of the sets designated as satellite group) to monitor possible self-recovery effects, the remaining animals (40) were evenly distributed into 8 treatment groups of 5 rats each and treated as in Table 1.

Treatments were done once daily via oral intubation between 9.00 and 10.00 a.m. to minimize possible diurnal effects. A transition period of 24 h was observed between the two subsequential treatment periods in both pre- and posttreatment groups.

### 2.7. Serum Preparation and Kidney Isolation

Forty-eight hours after the last treatment in each case, the rats were humanely euthanized under halothane anaesthetization and blood was collected via cardiac puncture into plain sample bottles. For serum preparation, the blood was allowed to clot for 10 min and subsequently centrifuged (Beckman and Hirsch, Burlington, IO, USA) at 3,000 ×g for 15 minutes. Serum was carefully aspirated and used for kidney function tests. The rats were also immediately dissected and the kidneys were diligently harvested, blotted with clean tissue paper, cleaned of fat, and weighed and the relative kidney-body weight ratios (RKW) were evaluated. The left kidney was thereafter sliced into two portions with one of the portions homogenized in Tris-HCl buffer (0.05 mol/L Tris-HCl and 1.15% KCl, pH 7.4) for antioxidant analyses, while the other was used for histological examination.

### 2.8. Biochemical Analyses

#### 2.8.1. Kidney Function Parameters

Following the procedures outlined in the assay kits, kidney function parameters were determined. Serum concentrations of creatinine, blood urea nitrogen, uric acid, potassium, sodium, and calcium were evaluated. Creatinine clearance rate (CCR) was estimated as earlier reported [22].

### 2.9. Antioxidant and Oxidative Stress Assays

#### 2.9.1. Reduced Glutathione (GSH) and Oxidized Glutathione (GSSG)

The procedure described by Ellman [23] was employed to determine the level of GSH in the homogenate. Briefly, 1.0 mL of the homogenate was added to 25% trichloroacetic acid (1 mL) and the precipitate was removed by centrifugation at 5,000 ×g for 10 min. Supernatant (0.1 mL) was added to 2 mL of 0.6 mM 5,5′-dithiobis-2-nitrobenzoic acid (DTNB) prepared in 0.2 M sodium phosphate buffer (pH 8.0). The absorbance of the yellow-colored complex was thereafter read at 420 nm and the extrapolated values from the standard calibration curve were expressed as the homogenate concentrations of GSH.

For GSSG level estimation, the described method of Hissin and Hilf [24] was adopted. The homogenate (50 *μ*L) was mixed with 20 *μ*L of 0.04 M* N*-ethylmaleimide (NEM) to prevent oxidation of GSH to GSSG. The mixture was subsequently incubated at room temperature for 30 min prior to consecutive addition of 0.3 M Na_2_HPO_4_ solution (1.68 mL) and 250 *μ*L of DTNB reagent. The absorbance of the resulting mixture was thereafter read at 420 nm as concentration of GSSG in the homogenate expressed in nmol/mg protein.

### 2.10. Lipid Peroxidation Products

The homogenate levels of lipid peroxidation products (conjugated dienes, lipid hydroperoxides, and malondialdehyde) were estimated as reported by Reilly and Aust [25].

### 2.11. Protein Carbonyl and Advanced Oxidation Protein Product (AOPP)

The method of Levine et al. [26] based on the reaction of carbonyl compounds with 2,4-dinitrophenyl hydrazine for 1 h and subsequent precipitation with 20% trichloroacetic acid was employed to determine the concentration of protein carbonyl in the renal homogenate. The released carbonyl compounds were measured spectrophotometrically at 380 nm and expressed as nmol/mg protein of the homogenate.

For the AOPP assay, the kidney homogenate (2 mL) was centrifuged at 2500 ×g for 10 min at 40°C. The resulting supernatant was thereafter added to a reaction mixture containing 50% acetic acid and 1.16 mol/L potassium iodide in phosphate buffered saline solution. The absorbance was read at 340 nm and the concentration of AOPP determined from the extrapolated standard curve of serially diluted AOPP standard solution using 500 *μ*mol/L chloramines stock [27].

### 2.12. Fragmented DNA

The quantity of fragmented DNA in the kidney homogenates was determined using standard protocols [28]. In brief, kidney homogenate was centrifuged at 15,000 ×g, for 15 min at 4°C. While the supernatant was aspirated and treated with 10% trichloroacetic acid (1.50 mL), the resulting pellet was treated with 5% trichloroacetic acid (0.65 mL). The reaction mixture in each case was kept refrigerated (4°C) to precipitate overnight before centrifuging at 2500 ×g for 10 min. Each reaction mixture was subsequently boiled at 100°C for 15 min, cooled to room temperature, and further centrifuged at 2500 ×g for 5 min. Exactly 0.5 mL of the supernatant content was treated with diphenylamine reagent (1 mL) and incubated at 37°C for 4 h. Absorbance readings for both the treated supernatant and pellet were taken at 600 nm using a spectrophotometer and the % fragmented DNA was calculated using the expression:

(1) $$\begin{array}{c} \%\;\;Fragmented\;\;DNA=100\times \left(\;\frac{A_{s}}{A_{s}+A_{p}}\;\right), \end{array}$$

where *A*_*s*_ and *A*_*p*_ represent absorbance of the supernatant and pellet, respectively.

### 2.13. Glutathione Peroxidase and Glutathione Reductase

Homogenate activities of glutathione peroxidase (GPx) and glutathione reductase (GRx) were also evaluated as per the manufacturer's instructions in the assay kits.

### 2.14. Superoxide Dismutase

The activity of superoxide dismutase (SOD) in the tissue homogenate was determined as outlined by Misra and Fridovich [29]. In brief, 0.2 mL of the homogenate was added to 2.5 mL of 0.05 M carbonate buffer (pH 10.2) to equilibrate before addition of freshly prepared 0.3 mM epinephrine (0.3 mL) to commence the reaction. The change in absorbance was measured at 480 nm at 30 s intervals for 150 s. One unit of enzyme activity is defined as 50% inhibition of the rate of autooxidation of pyrogallol as determined by changes in absorbance/min at 480 nm.

### 2.15. Catalase

The homogenate activity of catalase (CAT) activity was evaluated adopting the method of Aebi [30]. Exactly 50 *μ*L of the kidney homogenate was added to a cuvette containing 2 mL of phosphate buffer (pH 7.0) and 30 mM H_2_O_2_ (1 mL). Catalase activity was measured at 240 nm for 1 min using a spectrophotometer. The molar extinction coefficient (43.6 M/cm) of H_2_O_2_ was used to estimate catalase activity.

### 2.16. Histopathological Examination

Following previously reported standard protocol [31], the histopathological examination of the excised kidney was performed. Briefly, sliced portions of the kidney were washed in normal saline and fixed immediately in 10% buffered formalin solution for at least 24 h. They were further dehydrated with graded alcohol (50–100%) and subsequently processed in paraffin embedding using LEICA PT 1020 Automatic Tissue Processor. About 5 *μ*m thick section of each tissue was stained with hematoxylin and eosin and observed for possible histopathological infiltrations. Microscopic features of the kidneys of CSEAF- and vitamin C-treated rats were compared with both normal and nephrotoxic control groups. Based on the degree of derangements and severity of renal damage observed, the kidney sections were further evaluated and scored by an independent histopathologist on a 0 to 4 scale as follows:

0: normal and well-preserved renal architecture;
1: proximal convoluted tubules dilatation, focal granulovacuolar epithelial cell degeneration, and granular debris in not more than 1% of the tubular lumen;
2: epithelial necrosis and desquamation involving less than 50% of cortical tubules;
3: epithelial desquamation and necrosis involving more than 50% of proximal tubules;
4: complete or almost entire tubular necrosis.

### 2.17. Membrane Stabilizing Activity

#### 2.17.1. Preparation of Bovine Red Blood Cell Suspension

This was achieved following the method of Oyedapo et al. [32] with slight modification. Briefly, fresh bovine blood samples were collected into ethylenediaminetetraacetic acid (EDTA) bottles and centrifuged (Bench Centrifuge, Beckman and Hirsch, Burlington, IO, USA) at 3,000 ×g for 10 min. The supernatants (plasma and leucocytes) were carefully aspirated with a Pasteur pipette, while the packed red blood cells were washed five times with isotonic buffered solution (154 mM NaCl in 10 mM sodium phosphate buffer (pH 7.4)) and centrifuged (3000 ×g, 10 min) each time until the supernatants were clear. The resulting pellet was employed to prepare 2% (v/v) stock suspension of erythrocytes (RBC) that was subsequently used.

### 2.18. Hypotonic Solution-Induced Hemolysis

Earlier reported methods [32, 33] were adapted for this assay. In brief, 0.5 mL of stock erythrocyte (RBC) suspension was mixed with 4.5 mL of hypotonic solution (50 mM NaCl in 10 mM sodium phosphate buffered saline (pH 7.4)) containing 1.0 mL of either the fraction (0.25–2.0 mg/mL) or ibuprofen (standard drug (0.1 mg/mL)). For the control sample, 0.5 mL of RBCs was mixed with the hypotonic buffered saline alone. The resulting mixture in each case was incubated (20°C, 10 min) and subsequently centrifuged (3000 ×g, 10 min) prior to absorbance reading at 540 nm using a spectrophotometer (Beckman, DU 7400, USA). The percentage inhibition of either hemolysis or membrane stabilization was calculated using the following equation:

(2) $$\begin{array}{c} \%\;\;inhibition\;\;of\;\;hemolysis=100\times \left(\;\frac{{A_{c}-A}_{s}}{A_{c}}\;\right), \end{array}$$

where *A*_*c*_ is absorbance of control (hypotonic buffered saline solution alone) and *A*_*s*_ is absorbance of test sample in hypotonic solution.

### 2.19. Statistical Analysis

Degrees of protection conferred on the DNA, kidney function parameters, and inhibition of hemolysis by the fraction were expressed as percentages. Other results were subjected to one-way analysis of variance (ANOVA) using SPSS software package for windows (Version 16, SPSS Inc., Chicago, USA) and presented as mean ± standard error of mean (SEM) of five determinations. Significant difference between the treatment means was determined at 95% confidence level using Duncan's Multiple Range Test.

## 3. Results

### 3.1. Body and Relative Organ Weight

Data obtained with respect to body weight gain revealed significant (*p* < 0.05) reduction in the body weight of the nephrotoxic rats (APAP-treated) compared to control (Table 2). In contrast, when compared with the nephrotoxic group, the CSEAF pre- and posttreated groups had significantly (*p* < 0.05) higher weight gain with the effect elicited by the fraction administered at 200 mg/kg b.w. competing favorably with vitamin C. However, only marginal variation was observed in this parameter between the 200 mg/kg b.w. fraction-pretreated groups and those placed on 200 mg/kg b.w. of the fraction alone (Table 2). In addition, while there was marked reduction in the RKW of the APAP-treated animals, those of the fraction and vitamin C-administered groups were only marginally different from the normal control group (Table 2).

### 3.2. Kidney Function Indices

**Figure 1:**
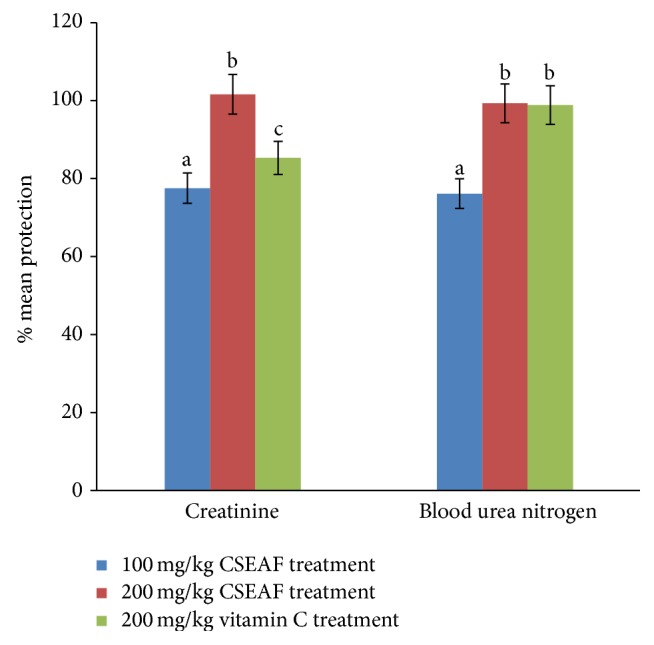
Mean percentage protection offered by* Zea mays*,* Stigma maydis* and vitamin C against acetaminophen-induced renal injury as assessed by serum creatinine and blood urea nitrogen. The percent protection was calculated as follows: 100 × (values of APAP treatment − values of test)/(values of APAP treatment − values of control). ^abc^Bars with different superscripts for each parameter are significantly different (*p* < 0.05). CSEAF: corn silk ethyl acetate fraction.

Significantly (*p* < 0.05) elevated serum levels of creatinine, urea, uric acid, sodium, and potassium were observed in the APAP-administered group when compared with the normal control (Tables 3 and 4). However, treatments with the fraction for 14 days dose-dependently and significantly (*p* < 0.05) prevented and extenuated the APAP-mediated increases in these parameters with most prominent effects elicited at the highest investigated dose in the pretreated groups. This is also consistent with the increased CCR and serum calcium levels observed in the fraction-supplemented rats as compared to the significantly (*p* < 0.05) reduced value for this parameter in the nephrotoxic rats (Tables 3 and 4). There were no evidences of nephrotoxic tendencies in the animals given 200 mg/kg b.w. of the CSEAF alone, as they compared favorably with the control for these parameters. However, the marked improvements observed in the fraction-treated animals were not evident in the satellite self-recovery group whose assayed parameters were essentially those of the nephrotoxic animals. The degree of protection conferred on the serum levels of creatinine and blood urea nitrogen by CSEAF treatments is presented in Figure 1. While treatments with 200 mgkg^−1^ b.w. CSEAF compared favorably with vitamin C for overall modulation of blood urea nitrogen, it elicited a better and most prominent effect on creatinine metabolism (Figure 1).

### 3.3. Antioxidants and Oxidative Stress Markers

#### 3.3.1. Nonenzymic Antioxidants and Oxidative Stress Markers

**Figure 2:**
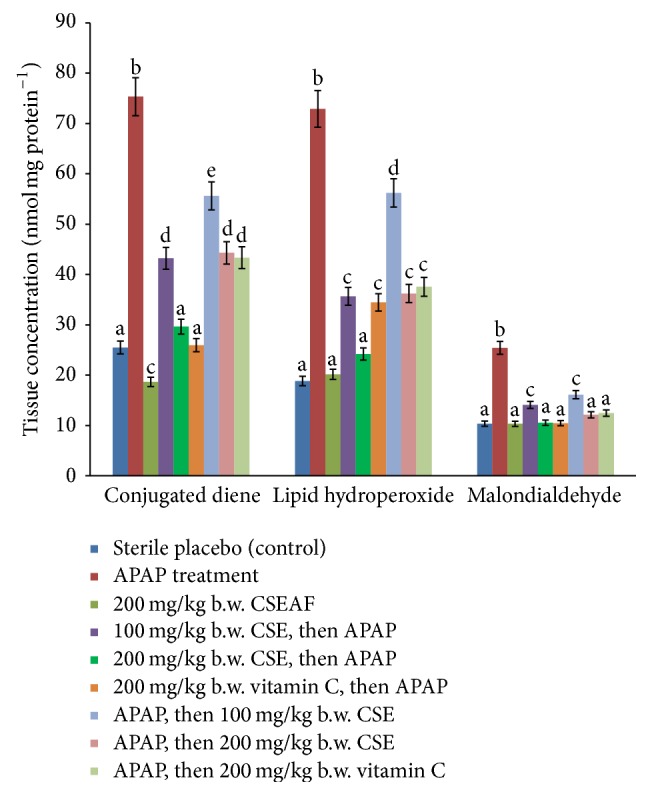
Effect of* Zea mays*,* Stigma maydis* ethyl acetate fraction on tissue concentrations of conjugated dienes, lipid hydroperoxides, and malondialdehyde of acetaminophen-treated rats. Values are mean ± standard error of mean (SEM) of five determinations. ^abcde^Bars with different superscripts for each parameter are significantly different (*p* < 0.05). APAP: acetaminophen and CSEAF: corn silk ethyl acetate fraction.

The effects of 14-day treatment with CSEAF on the nonenzymic antioxidant status and oxidative stress markers of the experimental rats are presented in Table 5 and Figure 2. The APAP-induced significant (*p* < 0.05) reduction in the level of GSH and the elevations in the levels of GSSG, protein carbonyls, AOPP, fragmented DNA, malondialdehyde, conjugated dienes, and lipid hydroperoxides were significantly (*p* < 0.05) and dose-dependently normalized in the CSEAF-treated animals. Although the fraction (at 200 mgkg^−1^ b.w.) compared well with reference drug (vitamin C) in both pre- and posttreatment modes, better results comparable to those of the control were observed in the fraction-pretreated group. However, while other nonenzymic antioxidant parameters were not significantly altered by treatment with 200 mgkg^−1^ b.w. of CSEAF alone, the homogenate level of GSH was significantly increased when compared with the control (Table 5).

#### 3.3.2. Enzymic Antioxidants

Kidney homogenate activities of GRx, GPx, SOD, and CAT were significantly (*p* < 0.05) induced by CSEAF in a concentration-dependent manner in the two treatment models. These inductions markedly (*p* < 0.05) improved the observed APAP-mediated reduction in their activities and at the highest investigated dose the effect compared well with that of vitamin C (Table 6).

### 3.4. Histopathological Investigation

**Figure 3:**
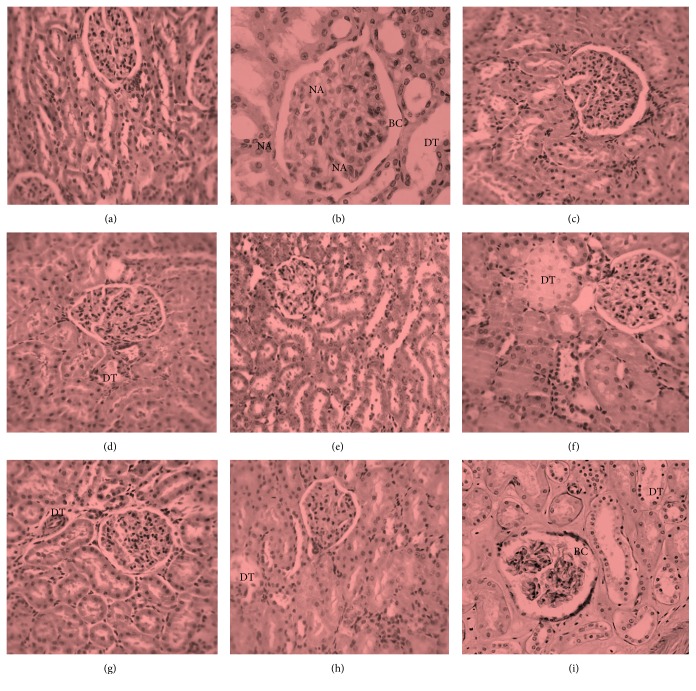
Kidney micrographs (×400, hematoxylin and eosin stained) of (a) control rat, (b) nephrotoxic rat, (c) CSEAF (200 mg/kg b.w.) treated rat, (d) nephrotoxic rat pretreated with CSEAF (100 mg/kg b.w.), (e) nephrotoxic rat pretreated with CSEAF (200 mg/kg b.w.), (f) nephrotoxic rat pretreated with vitamin C (200 mg/kg b.w.), (g) nephrotoxic rat posttreated with CSEAF (100 mg/kg b.w.), (h) nephrotoxic rat posttreated with CSEAF (200 mg/kg b.w.), and (i) nephrotoxic rat posttreated with vitamin C (200 mg/kg b.w.). CSEAF: corn silk ethyl acetate fraction, BC: Bowman's capsule showing leukocyte infiltration, DT: dilated proximal tubule, and NA: necrotic area.

Macroscopic examination of kidneys from the control group revealed that they were essentially normal with characteristic fine texture and dark maroon appearance. While kidneys from the fraction-administered animals showed mild spots of brown color changes, those of the APAP-intoxicated animals revealed color changes from maroon to brown with characteristic uneven texture. Detailed histoarchitectural examination of the kidney sections of the control and 200 mgkg^−1^ b.w. CSEAF groups showed no histological derangements, as evidenced by the normal and well-preserved renal architecture with characteristic intact glomeruli and tubules (Figures 3(a) and 3(c)). In contrast, kidney sections from the APAP-administered group exhibited altered architecture with extensive destruction of glomeruli and tubular structures, as demonstrated by marked necrotic areas (Figure 3(b)). Hypercellularity in Bowman's capsule indicating leukocyte infiltration, glomerular atrophy, and dilated proximal tubules with loss of the cellular boundary and brush border were also evident (Figure 3(b)). Treatment with CSEAF at the investigated doses in the pre- and posttreatment modes showed dose-dependent protective and ameliorative capabilities in the kidneys that compared favorably with that of vitamin C (Figures 3(d)–3(i)). This was evident by the less severe tubular and glomerular damage, with the best and most prominent effect observed in the 200 mgkg^−1^ b.w. dose treated groups (Figures 3(e) and 3(h)). Furthermore, histopathological scoring of the kidney sections of CSEAF-treated groups showed that the obvious epithelial desquamation and tubular necrosis present in the kidney sections of the APAP-intoxicated animals were significantly and dose-dependently assuaged in a manner comparable to the vitamin C-treated animals (Table 7).

### 3.5. Membrane Stabilization

**Figure 4:**
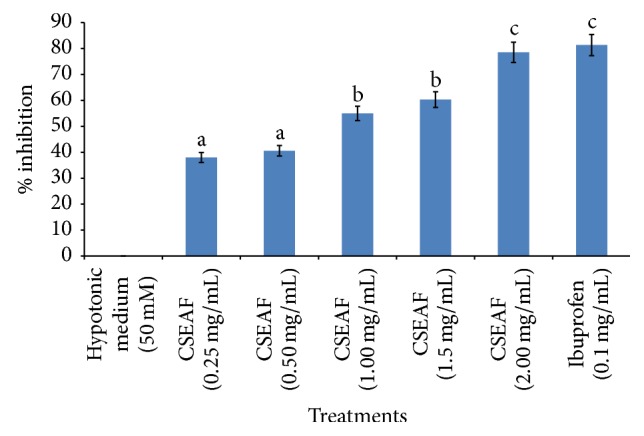
Effect of* Zea mays*,* Stigma maydis* ethyl acetate fraction on hypotonic solution-induced hemolysis of bovine erythrocyte membrane. Values are mean ± standard error of mean (SEM) of three determinations. ^abc^Bars with different superscripts for the parameter are significantly different (*p* < 0.05). CSEAF: corn silk ethyl acetate fraction.

The result of membrane stabilizing activity of CSEAF is shown in Figure 4. Treatment with the fraction dose-dependently protected bovine RBC against hypotonic solution-induced infiltrations. The elicited effect at a 2 mg/mL dose of the fraction compared favorably with ibuprofen (0.1 mg/mL), standard drug used in this study.

## 4. Discussion

Acetaminophen-mediated oxidative nephrotoxicity has been well documented and is characterized by morphologic and functional evidence of proximal tubular injury in humans and experimental animals [34, 35]. Acute and chronic renal failures in the course of therapeutic APAP administration have also been described in alcoholics and case-control studies [36]. Although molecular studies have linked APAP renal proximal tubular damage to translocation of GADD153 (growth arrest- and DNA damage-inducible gene 153) to the nucleus and subsequent proteolysis of caspase-12 [37], involvement of a reactive intermediate metabolite, N-acetyl-*p*-benzoquinone imine (NAPQI), cannot be excluded from renal damage [21]. NAPQI arylates selenium binding protein and glutamine synthetase in the S3 segment of the proximal tubule with consequential depletion of GSH [38]. This subsequently results in autooxidation of renal macromolecules (lipids, proteins, and DNA) with associated tubular cell necrosis. Tubular cell loss is an important feature of both acute renal failure and chronic renal disease [35] and is accompanied by concomitant increased serum concentrations of creatinine, urea, uric acid, and electrolyte imbalance. Creatinine, urea, and uric acid are major catabolic products of muscle, protein, and purine metabolism, respectively, and their serum concentrations give clues to the functional capacity of the nephrons at the glomerular and tubular levels [39]. These waste products (urea and creatinine) are passed into the blood stream for removal by the kidneys and their increased level in blood is a direct indication of renal dysfunction [40].

In this study, the increased serum concentrations of creatinine, blood urea nitrogen, and uric acid coupled with the attenuated CCR in the APAP-intoxicated animals may be indicative of renal injury and cell necrosis resulting from formation of NAPQI in excess of GSH detoxification ability. This is consistent with previous studies [21, 34], where APAP administration proved toxic to renal tubular cells. However, the significant and dose-dependent reversal in the levels of these parameters in the CSEAF-treated rats suggests that CSEAF was able to prevent or extenuate the deleterious influence of APAP. This observation also indicates that CSEAF at the investigated doses could preserve renal functions and delay progression of renal pathological conditions.

The most common cause of electrolyte imbalance or disturbance is associated with renal failure [40]. Calcium ions play a vital role in muscle contraction and serves as an intracellular second messenger for hormones. Hypocalcaemia is most commonly found in terminal stages of chronic generalized renal failure. Hence, the low level of calcium ion in the serum of APAP-administered rats may be associated with derangement of renal function resulting from interference with ions transport across the renal tubules [41]. The improvement observed in the calcium concentration in CSEAF-treated animals is a probable indication of its nephroprotective tendency. Sodium and potassium are the major extracellular and intracellular cations, respectively, in a living system. Sodium regulates the total amount of water in the body and its transmission across cells plays roles critical to body functions while adequate levels of potassium ions are essential for normal cell function. Many processes in the body, especially in the nervous system, muscles, and renal selective reabsorption, require electrical signals for communication. The movement of these ions is critical in generation of electrical signals [42]. In this study, the concentration-related significant normalization of APAP-mediated increases in serum levels of sodium and potassium ions in the fraction-administered rats is suggestive of its ability to maintain the levels of water and sodium at optimum equilibrium, thereby enhancing selective reabsorption capability of the nephron. Since membrane integrity is vital to signalling, the effect observed may also be due to the potential of the fraction to maintain membrane integrity of the kidney cells. This agrees with a previous report [43] where treatment with plant extracts reversed an acetaminophen-induced electrolyte imbalance in experimental animals.

Changes in the body weight of animals have been used to predict the nature and extent of drug-induced toxicity and may give important information on their overall health status [44]. It could also dictate the impact of the drug on the overall growth and developmental metabolism of the animals. Therefore, the observed reduction in the body weight of the APAP-treated animals may imply possible impairment in growth-linked metabolic processes. That the CSEAF-administered animals had relatively normal and marginal body weight gain is an obvious indication of the tendency of the fraction to aid normal metabolism and sustain growth and developmental mechanisms in the animals. This could be attributed to enhanced appetite in the animals that may be ascribed to the phytoconstituents in CSEAF as previously reported for the crude aqueous extract of CS [45]. The effect of fraction on the body weight of the animals not only was further supported by the significant weight gain in the rats placed on 200 mgkg^−1^ b.w. dose of CSEAF alone, but also lent credence to the nonnephrotoxic effect of the fraction. This observation is consistent with the finding of Abdul Hamid et al. [46], where administration of standardized leaf extract of* Zingiber zerumbet* was linked to both mean body weight gain and nephroprotective effects in experimental animals. The relative organ weight in pharmacological studies is imperative to understanding crucial treatment-induced organ weight variations in animals [47]. While an increase in the relative organ weight may depict either inflammation or increased secretory ability of the organ, a reduction could be informative of cellular shrinkage. In addition to defining toxicity as pathological changes observed in the organs of interest, the relative organ weight could also be suggestive of organ swelling, atrophy, or hypertrophy [48]. In this study, the sustained kidney weights in all the fraction-administered animals relative to the significantly reduced kidney weight in the APAP-intoxicated rats could imply that the constriction of renal tubular cells caused by the ravaging oxidative insults of APAP was well prevented or extenuated by the fraction. This suggests that CSEAF could protect renal tubular cells against oxidative routs at the tested doses and was closely supported by the histopathological findings where the kidney sections of fraction-treated animals revealed distinct and well-preserved histoarchitectural features.

Studies have implicated NAPQI formation and oxidative stress in APAP-induced nephrotoxicity [49, 50], and their overwhelming effects result in significantly impaired and insufficient levels of both enzymic and nonenzymic antioxidant defense mechanisms in the body [51]. Hence, the observed reduction in the renal level of GSH of APAP-intoxicated rats in this study might be due to depletion of GPx and GR, as well as formation of NAPQI that exceeds GSH detoxification capacity [49]. Also, the APAP-mediated elevation in GSSG levels may be ascribed to either GSH autooxidation or its mobilization towards GPx formation. The reduction in the ratio of GSH to GSSG caused by APAP administration reveals possible oxidative damage on the renal tubular cells. However, the significantly and dose-dependently increased GSH level coupled with the corresponding high GSH: GSSG ratio and low GSSG levels in the kidneys of the CSEAF-treated rats is suggestive of the inherent antioxidative effect of the fraction and further supports that it offered a considerable level of nephroprotection. Palani et al. [52] also reported similar improvement on the nonenzymic antioxidant status in APAP-intoxicated rats following treatment with* Pimpinella tirupatiensis* ethanolic extract.

An elevated level of malondialdehyde in tissues is an obvious indication of cellular damage due to lipid peroxidation resulting from malfunctioning of the antioxidant defense system [53]. Furthermore, APAP has been linked with lipid peroxidation and may facilitate elevated level of peroxidized products (conjugated dienes, lipid hydroperoxides, and malondialdehydes) in nephrotoxicity [46]. Therefore, the significant elevation in the levels of these products could be indicative of uncontrolled oxidative attacks of APAP's reactive metabolites and ROS on membrane-bound lipids. This might have disrupted membrane fluidity as well as modified and inflicted functional loss on the proteins and DNA of the renal tubular cells. The attenuation of APAP-enhanced increases in these peroxidative products by the CSEAF could mean that it was able to offer a considerable level of protection to the renal membrane lipids. This may be adduced to the ability of the CSEAF to aid with detoxification of reactive metabolites, which could have initiated and propagated peroxidation of membrane-bound polyunsaturated lipids of the tubular cells. It may also suggest that the fraction is rich in phytonutrients capable of stabilizing the cellular membranes of renal tubules against oxidative onslaughts of APAP. In the same vein, unguided influence of ROS can trigger protein autooxidation with consequential formation of protein carbonyls and AOPP [54]. These protein oxidative products have been employed as invaluable markers to ascertain the degree of oxidant-mediated protein damage in cells [55]. In this study, the significant increase in the tissue concentrations of these markers in the nephrotoxic animals relative to the fraction-treated rats could be attributable to the capacity of NAPQI to arylate selenium binding protein and glutamine synthetase in the S3 segment of the proximal tubule, thereby facilitating autooxidation of renal proteins [38]. This may also imply covalent binding of NAPQI to mitochondrial proteins which resultantly induced nitrate ion formation that incapacitates the Ca^2+^ pump of the renal membrane. Incapacitation of the Ca^2+^ pump further hinders mitochondrial function and ATP production which could have enhanced the observed elevated levels of AOPP and carbonyl product [54, 55]. The pre- and posttreatments with CSEAF reversed this trend which is a further attestation to its possible potential to incapacitate NAPQI, nitrate ions, and other reactive metabolites through enhancement and fortification of the antioxidant defense mechanisms of the renal tubular cells. Our findings are in agreement with previous assertions [46] that linked restoration to normal of APAP-mediated elevated levels of renal protein-oxidized products with seven-day treatment with ethyl acetate extract of* Zingiber zerumbet*.

Similar to protein-oxidized products, calcium ion accumulation and hydroxyl radical mediated oxidative damage are important events in the pathogenesis of DNA fragmentation. These events promote either tissue necrosis or carcinogenesis, which subsequently results in cell death [56]. Thus, the significantly increased level of fragmented DNA in the kidneys of APAP-intoxicated rat is indicative of either genotoxicity or probable initiation of carcinogenesis. Jaeschke and Bajt [57] have earlier reported a similar increase in the level of damaged DNA due to APAP administration. The CSEAF-enhanced attenuation in the level of fragmented DNA in the kidneys of APAP-treated rats is a tenable fact that the fraction is endowed with antioxidative and antigenotoxic attributes. The CSEAF might have mimicked an antigenotoxic agent, thereby facilitating DNA repair or synthesis system [58].

During renal damage, superoxide radicals are formed at the site of injury and if their accumulation exceeds the body's antioxidant capacity it could overwhelm the defensive activities of SOD and CAT, thereby aggravating the severity of existing damage [52]. Therefore, exogenous optimization of preventive (CAT, GPx) and chain-breaking (SOD, GRx) enzymic antioxidants that subsequently increase cellular GSH content is not only imperative to annihilating catastrophic influence of free radicals/ROS but also crucial to stalling ravaging impacts of drug-induced oxidative stress. In this study, the decreased tissue activities of the assayed antioxidant enzymes (SOD, CAT, GRx, and GPx) could be due to their excessive mobilization towards detoxification of NAPQI and ROS during APAP-induced nephrotoxicity. This might have led to haphazard oxidative attack on cellular macromolecules that consequently results in necrosis [59]. This report is in agreement with the findings of Aslam et al. [60] and Kadir et al. [61], where the authors also studied drug-mediated renal damage in rats. They noted similar reductions in the activities of ROS detoxifying enzymes that were associated with the formation of reactive metabolites and ROS. Thus, the dose-dependent auspicious reversion of the APAP-induced reduction in the activities of these detoxifying enzymes by the CSEAF is indicative of its antioxidative activity. This could either be attributed to the tendency of the fraction to scavenge NAPQI and ROS or enhance tissue activity of ROS detoxifying enzymes.

In addition to complementing biochemical analyses, histopathological examination of kidney sections may provide invaluable information on how pharmacologically potent an agent is against renal damage. The significant alterations in glomerular structure, the thickening of the glomerular basement membrane, widening of the filtration slits, basal infolding patterns with the presence of cytoplasmic vacuolation, and the increase in collagen deposition around the tubules as observed in the kidney sections of APAP-intoxicated rats could be responsible for their impaired renal function. The consequently decreased glomerular filtration rate with associated elevated serum levels of urea and creatinine was evident in the present study and agreed with a previous report [62]. However, the apparently repudiated oxidative threats inflicted by APAP on the architectural features of renal tubular cells in the fraction pre- and posttreated rats suggest that the CSEAF offered a significant degree of protection and stabilization on the overall histoarchitectural integrity of the kidneys. In fact, the preservation of the tubular cells and architectural organization of some of the kidney sections was almost completely normalized with an increasing number of viable cells. The effects noticed compared favorably with that of vitamin C and are consistent with the results of the biochemical investigations performed in this study. Our observations agree with previous reports [21, 46, 63], where recovery from APAP-mediated derangements towards normalization of serum kidney function parameters and renal histological architecture was attributed to treatment with plant extracts.

During inflammatory events, lysosomal enzymes and hydrolytic constituents are released from phagocytes into the extracellular space. This release consequently inflicts injuries on the surrounding organelles and tissues as well as aggravating the severity of any existing infection [33]. The exposure of an erythrocyte to injurious substances like hypotonic medium and heat results in lysis of its membrane with associated hemolysis and autooxidation of hemoglobin. The autooxidation and hemolysis are direct consequences of the vulnerability of the cells to secondary insults of free radical chain reactions [64]. This hemolytic effect is closely linked to excessive accumulation of fluid within the cell which consequently results in membrane disorientation and disruption. This notion is consistent with the observation that the breakdown of biomembranes generates free radicals that invariably enhance cellular damage as normally observed in renal tubular oxidative damage [61]. Medicinal plants with good antioxidative and anti-inflammatory attributes have been reported to provide succor either by annihilating the ravaging activity of lysosomal enzymes or by enhanced stabilization of biomembranes via maintenance of membrane fluidity and ion gradients [32, 65]. This was evidently displayed in this study with the CSEAF at the highest investigated dose conferring a membrane stabilization potential of 78.55% against hypotonic solution infiltration on bovine erythrocytes. This may be ascribed to either the capability of the fraction to bind tenaciously to the erythrocyte membranes with concomitant prevention of deleterious assaults of the hypotonic solution or its potential in promoting dispersion by mutual repulsion of charges involved in the hemolysis of RBCs. While studies have shown flavonoids to exert stabilizing effects on lysosomes [33], tannin and saponins have been reported as being capable of binding cations, thereby stabilizing the erythrocyte membrane [66]. In addition to the various antioxidative phytoconstituents revealed by GC-MS analysis of CS, alkaloids, sterols, phenols, tannins, flavonoids, and saponins have also been qualitatively and quantitatively evaluated in its crude aqueous extract [67]. Therefore, the remarkable RBC membrane stabilization activity with good protection against hypotonic solution-induced lysis elicited by the CSEAF in this study may be attributed to the presence of these phytochemicals. This might have also enhanced restoration to normal of APAP-perturbed alterations in the assayed biochemical indices and preserved the membrane integrity of renal tubular cells as evidently revealed in both pre- and posttreatment groups in this study.

## 5. Conclusion

The reduction of oxidative onslaughts posed by APAP via treatments with the ethyl acetate fraction of corn silk is a manifestation of its capabilities to preserve renal function and delay progression of renal pathological conditions to end stage disease/death. The engendered nephroprotective effect by the fraction could, in rats, be ascribed to its antioxidative and membrane stabilization potential. This is achieved by facilitating detoxification of APAP-mediated nephrotoxicity via induction of ROS detoxifying enzymes, thereby stalling autooxidation of cellular macromolecules and renal tubular damage. Though the effects were prominently exhibited in the fraction-pretreated groups, the overall effects elicited in both treatment groups were remarkable and indicative of an excellent candidate for the management of drug-induced renal oxidative disorders.

## Figures and Tables

**Table 1:** 

Groups	Designation	Treatments
1	Control	Given sterile placebo.
2	Nephrotoxic rats in two sets	Animals induced with nephrotoxicity and not treated.
3	CSEAF-treated only	Given 200 mgkg^−1^ b.w. of CSEAF only for 14 days.
4, 5, and 6	Pretreatment	Pretreated with CSEAF (100 and 200 mgkg^−1^ b.w.) and vitamin C (200 mgkg^−1^ b.w.), respectively, for 14 days prior to nephrotoxicity induction.
7, 8, and 9	Posttreatment	Nephrotoxic rats posttreated, respectively, with the fraction (100 and 200 mgkg^−1^ b.w.) and vitamin C (200 mgkg^−1^ b.w.) for 14 days.

**Table 2:** Effect of *Zea mays*, *Stigma maydis* ethyl acetate fraction on the body weight changes and relative organ weights of acetaminophen-treated rats (*n* = 5, mean ± SEM).

Treatments	Weight changes			Kidney weight (g)	RKW (g/100 g b.w.)
	Initial (g)	Final (g)	% weight gain		
Sterile placebo (control)	210.22 ± 0.90	224.01 ± 0.89	6.16^a^	1.61 ± 0.01^a^	0.72^a^
APAP treatment	220.12 ± 0.77	215.00 ± 0.99	(2.34^b^)	0.97 ± 0.01^b^	0.45^b^
200 mg/kg b.w. CSEAF	200.05 ± 0.54	220.01 ± 0.86	9.07^c^	1.69 ± 0.01^a^	0.77^a^
100 mg/kg b.w. CSEAF, then APAP	212.21 ± 0.75	223.09 ± 0.76	4.88^a^	1.58 ± 0.02^a^	0.71^a^
200 mg/kg b.w. CSEAF, then APAP	206.15 ± 0.45	227.31 ± 0.69	9.31^c^	1.73 ± 0.01^a^	0.76^a^
200 mg/kg b.w. vitamin C, then APAP	215.05 ± 0.39	228.19 ± 0.50	5.76^a^	1.64 ± 0.02^a^	0.72^a^
APAP, then 100 mg/kg b.w. CSEAF	200.15 ± 0.31	205.99 ± 0.56	2.84^d^	1.49 ± 0.02^a^	0.73^a^
APAP, then 200 mg/kg b.w. CSEAF	219.02 ± 0.97	232.00 ± 0.67	5.60^a^	1.72 ± 0.01^a^	0.74^a^
APAP, then 200 mg/kg b.w. vitamin C	221.09 ± 0.75	227.23 ± 0.45	2.70^d^	1.63 ± 0.01^a^	0.72^a^

Values bearing different superscripts along the same column for each parameter are significantly different (*p* < 0.05).

Parenthesis signifies reduced value for the parameter. RKW: relative kidney-body weight.

**Table 3:** Effect of *Zea mays*, *Stigma maydis* ethyl acetate fraction on serum concentrations of some kidney function parameters of acetaminophen-treated rats (*n* = 5, mean ± SEM).

Treatments	Creatinine (mg/dL)	BUN (mg/dL)	Uric acid (mg/dL)	CCR (mL/min)
Sterile placebo (control)	0.63 ± 0.02^a^	15.66 ± 0.17^a^	4.09 ± 0.01^a^	10.48 ± 0.01^a^
APAP treatment	2.50 ± 0.04^b^	49.00 ± 0.26^b^	15.99 ± 0.02^b^	2.64 ± 0.01^b^
200 mg/kg b.w. CSEAF	0.62 ± 0.01^a^	14.99 ± 0.43^a^	4.10 ± 0.03^a^	10.65 ± 0.02^a^
100 mg/kg b.w. CSEAF, then APAP	0.99 ± 0.03^c^	22.11 ± 0.23^c^	8.11 ± 0.09^c^	6.67 ± 0.01^c^
200 mg/kg b.w. CSEAF, then APAP	0.57 ± 0.02^a^	15.99 ± 0.13^a^	4.51 ± 0.03^a^	11.58 ± 0.02^a^
200 mg/kg b.w. vitamin C, then APAP	0.56 ± 0.02^a^	16.00 ± 0.12^a^	4.51 ± 0.09^a^	11.79 ± 0.01^a^
APAP, then 100 mg/kg b.w. CSEAF	1.11 ± 0.03^c^	25.12 ± 0.18^c^	8.99 ± 0.06^c^	5.95 ± 0.02^c^
APAP, then 200 mg/kg b.w. CSEAF	0.63 ± 0.05^a^	15.79 ± 0.12^a^	9.01 ± 0.06^c^	10.48 ± 0.01^a^
APAP, then 200 mg/kg b.w. vitamin C	1.25 ± 0.01^c^	16.09 ± 0.23^a^	8.88 ± 0.04^c^	5.28 ± 0.01^c^

^abc^Values with different superscripts for each parameter are significantly different (*p* < 0.05). APAP: acetaminophen, CSEAF: corn silk ethyl acetate fraction, BUN: blood urea nitrogen, and CCR: estimated creatinine clearance rate.

**Table 4:** Effect of *Zea mays*, *Stigma maydis* ethyl acetate fraction on serum concentrations of selected electrolytes of acetaminophen-treated rats (*n* = 5, mean ± SEM).

Treatments	Sodium (mEq/L)	Potassium (mEq/L)	Calcium (mg/dL)
Sterile placebo (control)	135.44 ± 1.35^a^	3.50 ± 1.01^a^	8.95 ± 1.01^a^
APAP treatment	338.22 ± 1.65^b^	10.11 ± 1.02^b^	3.33 ± 1.00^b^
200 mg/kg b.w. CSEAF	145.01 ± 1.21^a^	3.59 ± 1.00^a^	9.83 ± 1.02^a^
100 mg/kg b.w. CSEAF, then APAP	198.22 ± 1.11^c^	5.55 ± 1.02^c^	5.99 ± 1.01^c^
200 mg/kg b.w. CSEAF, then APAP	138.94 ± 1.26^a^	3.75 ± 1.06^a^	9.01 ± 1.00^a^
200 mg/kg b.w. vitamin C, then APAP	143.12 ± 1.70^a^	3.55 ± 1.01^a^	9.17 ± 1.01^a^
APAP, then 100 mg/kg b.w. CSEAF	226.21 ± 1.13^d^	7.09 ± 1.01^d^	6.99 ± 1.02^d^
APAP, then 200 mg/kg b.w. CSEAF	200.12 ± 1.42^c^	5.99 ± 1.00^c^	8.99 ± 1.02^a^
APAP, then 200 mg/kg b.w. vitamin C	139.01 ± 1.22^a^	3.79 ± 1.02^a^	9.15 ± 1.01^a^

^abcd^Values with different superscripts for each parameter are significantly different (*p* < 0.05). APAP: acetaminophen and CSEAF: corn silk ethyl acetate fraction.

**Table 5:** Effect of *Zea mays*, *Stigma maydis* ethyl acetate fraction on the levels of nonenzymic antioxidant system, protein-oxidized products, and fragmented DNA of acetaminophen-treated rats (*n* = 5, mean ± SEM).

Treatment	GSH (X)	GSSG (X)	GSH/GSSG	PC (X)	AOPP (Y)	F/DNA (%)
Sterile placebo (control)	35.11 ± 1.35^a^	0.20 ± 0.01^a^	175.55 ± 0.20^a^	3.72 ± 0.25^a^	195.98 ± 1.99^a^	10.12 ± 0.10^a^
APAP treatment	7.56 ± 1.09^b^	1.99 ± 0.02^b^	3.80 ± 0.08^b^	15.12 ± 0.22^b^	501.23 ± 1.59^b^	65.15 ± 0.19^b^
200 mg/kg b.w. CSEAF	25.02 ± 1.08^c^	0.12 ± 0.03^c^	208.50 ± 0.32^c^	3.66 ± 0.40^a^	190.23 ± 1.45^a^	11.75 ± 0.14^a^
100 mg/kg b.w. CSEAF, then APAP	30.19 ± 1.11^a^	0.21 ± 0.02^a^	143.29 ± 0.19^d^	8.00 ± 0.25^c^	275.99 ± 1.40^c^	30.16 ± 0.15^c^
200 mg/kg b.w. CSEAF, then APAP	31.01 ± 1.06^a^	0.16 ± 0.03^a^	193.81 ± 0.15^c^	4.67 ± 0.32^a^	193.01 ± 1.34^a^	12.01 ± 0.18^a^
200 mg/kg b.w. vitamin C, then APAP	36.11 ± 1.32^a^	0.19 ± 0.02^a^	190.05 ± 0.17^c^	3.89 ± 0.25^a^	200.45 ± 1.29^a^	11.21 ± 0.19^a^
APAP, then 100 mg/kg b.w. CSEAF	15.99 ± 1.11^d^	0.22 ± 0.01^a^	72.68 ± 0.15^e^	9.04 ± 0.22^c^	287.32 ± 1.88^c^	31.00 ± 0.10^c^
APAP, then 200 mg/kg b.w. CSEAF	16.21 ± 1.00^d^	0.19 ± 0.05^a^	85.32 ± 0.09^e^	9.14 ± 0.35^c^	203.19 ± 1.25^a^	12.09 ± 0.11^a^
APAP, then 200 mg/kg b.w. vitamin C	15.15 ± 1.72^d^	0.19 ± 0.05^a^	79.74 ± 0.10^e^	8.01 ± 0.29^c^	212.34 ± 1.67^a^	12.13 ± 0.15^a^

^abcde^Values with different superscripts for each parameter are significantly different (*p* < 0.05). APAP: acetaminophen, CSEAF: corn silk ethyl acetate fraction, GSH: reduced glutathione, GSSG: peroxidized glutathione, PC: protein carbonyl, AOPP: advanced oxidation protein product, F/DNA: fragmented DNA, X: nmol mg protein^−1^, and Y: *µ*mol mg protein^−1^.

**Table 6:** Effect of *Zea mays*, *Stigma maydis* ethyl acetate fraction on specific activities of enzymic antioxidant system of acetaminophen-treated rats (*n* = 5, mean ± SEM).

Treatments	Antioxidant enzymes (nmol min^−1^ mgprotein^−1^)			
	SOD	Catalase	Glutathione Rx	Glutathione Px
Sterile placebo (control)	43.12 ± 0.15^a^	33.99 ± 0.11^a^	56.11 ± 0.45^a^	123.15 ± 1.10^a^
APAP treatment	10.11 ± 0.10^b^	13.29 ± 0.12^b^	18.11 ± 0.23^b^	43.66 ± 1.15^b^
200 mg/kg b.w. CSEAF	59.21 ± 0.03^c^	45.35 ± 0.11^c^	55.19 ± 0.20^a^	144.11 ± 1.10^c^
100 mg/kg b.w. CSEAF, then APAP	32.12 ± 0.11^d^	22.76 ± 0.15^d^	30.21 ± 0.25^c^	102.31 ± 1.10^d^
200 mg/kg b.w. CSEAF, then APAP	41.24 ± 0.12^a^	32.99 ± 0.19^a^	53.99 ± 0.23^a^	125.03 ± 1.11^a^
200 mg/kg b.w. vitamin C, then APAP	42.07 ± 0.21^a^	33.09 ± 0.11^a^	56.04 ± 0.35^a^	100.19 ± 1.09^d^
APAP, then 100 mg/kg b.w. CSEAF	32.11 ± 0.12^d^	22.18 ± 0.13^d^	31.14 ± 0.31^c^	98.22 ± 1.15^d^
APAP, then 200 mg/kg b.w. CSEAF	31.99 ± 0.15^d^	31.00 ± 0.13^a^	55.09 ± 0.26^a^	120.11 ± 1.10^a^
APAP, then 200 mg/kg b.w. vitamin C	30.98 ± 0.20^d^	30.01 ± 0.12^a^	33.04 ± 0.35^c^	96.12 ± 1.12^d^

^abcd^Values with different superscripts for each parameter are significantly different (*p* < 0.05). APAP: acetaminophen, CSEAF: corn silk ethyl acetate fraction, SOD: superoxide dismutase, Rx: reductase, and Px: peroxidase.

**Table 7:** Histopathological grading of liver tissue sections of *Zea mays*, *Stigma maydis* ethyl acetate fraction-treated animals.

Treatments	Scores				
	0	1	2	3	4
Control	(5)	(0)	(0)	(0)	(0)
APAP treatment	(0)	(0)	(1)	(3)	(1)
200 mg/kg of CSEAF	(5)	(0)	(0)	(0)	(0)
100 mg/kg b.w. CSEAF, then APAP	(3)	(2)	(0)	(0)	(0)
200 mg/kg b.w. CSEAF, then APAP	(5)	(0)	(0)	(0)	(0)
200 mg/kg b.w. vitamin C, then APAP	(3)	(2)	(0)	(0)	(0)
APAP, then 100 mg/kg b.w. CSEAF	(2)	(2)	(1)	(0)	(0)
APAP, then 200 mg/kg b.w. CSEAF	(4)	(1)	(0)	(0)	(0)
APAP, then 200 mg/kg b.w. vitamin C	(3)	(2)	(0)	(0)	(0)

(*n* = 5; figure in parenthesis represents number of rats affected in the group). APAP: acetaminophen and CSEAF: corn silk ethyl acetate fraction.

## References

[1] Ozbek E. (2012). Induction of oxidative stress in kidney. *International Journal of Nephrology*.

[2] Silva F. G. (2004). Chemical-induced nephropathy: a review of the renal tubulointerstitial lesions in humans. *Toxicologic Pathology*.

[3] World Health Organization (WHO) (2014). *Track Records of Global Chronic Kidney Disorders: An Update for the Sub-Saharan African Countries 2011–2014*.

[4] National Kidney Foundation of South Africa (NKFS) Fact sheet on the prevalence of kidney disorders. http://www.nkf.org.za/kidney_disease.htm.

[5] Ahmad Q. Z., Jahan N., Ahmad G., Tajuddin G. (2014). An appraisal of nephroprotection and the scope of natural products in combating renal disorders. *Journal of Nephrology and Therapeutics*.

[6] World Health Organization (2005). *Preventing Chronic Disease: A Vital Investment*.

[7] Ullah N., Khan M. A., Khan T., Ahmad W. (2014). Nephroprotective potentials of *Citrus Aurantium*: a prospective pharmacological study on experimental models. *Pakistan Journal of Pharmaceutical Sciences*.

[8] Hamid N., Mahmoud R. (2013). Tubular kidney protection by antioxidants. *Iranian Journal of Public Health*.

[9] Grases F., March J. G., Ramis M., Costa-Bauzá A. (1993). The influence of *Zea mays* on urinary risk factors for kidney stones in rats. *Phytotherapy Research*.

[10] Sabiu S., O'Neill F. H., Ashafa A. O. T. (2016). *Zea mays, Stigma maydis* prevents and extenuates acetaminophen-perturbed oxidative onslaughts in rat hepatocytes. *Pharmaceutical Biology*.

[11] Guevara P., Pérez-Amador M. C., Zúñiga B., Snook M. (2000). Flavones in corn silks and resistance to insect attacks. *Phyton-International Journal of Experimental Botany*.

[12] Maksimović Z. A., Kovačević N. (2003). Preliminary assay on the antioxidative activity of *Maydis stigma* extracts. *Fitoterapia*.

[13] Kim K. A., Choi S.-K., Choi H.-S. (2004). Corn silk induces nitric oxide synthase in murine macrophages. *Experimental and Molecular Medicine*.

[14] Wang G.-Q., Xu T., Bu X.-M., Liu B.-Y. (2012). Anti-inflammation effects of corn silk in a rat model of carrageenin-induced pleurisy. *Inflammation*.

[15] Zhao W., Yin Y., Yu Z., Liu J., Chen F. (2012). Comparison of anti-diabetic effects of polysaccharides from corn silk on normal and hyperglycemia rats. *International Journal of Biological Macromolecules*.

[16] Ghada M., Eltohami M. S., Adurahman H. N., Mahmoud M. E., Mudawi I. (2014). *In Vitro* study of the effect of corn silk on glucose uptake by isolated rat hemi-diaphragm. *World Journal of Pharmaceutical Research*.

[17] Sukandar E. Y., Sigit J. I., Adiwibowo L. F. (2013). Study of kidney repair mechanisms of corn silk (*Zea mays* L. Hair)-binahong (*Anredera cordifolia* (Ten.) Steenis) leaves combination in rat model of kidney failure. *International Journal of Pharmacology*.

[18] Sepehri G., Derakhshanfar A., Zadeh F. Y. (2011). Protective effects of corn silk extract administration on gentamicin-induced nephrotoxicity in rat. *Comparative Clinical Pathology*.

[19] National Research Council (NRC) (2011). *Guide for the Care and Use of Laboratory Animals*.

[20] World Health Organization (1998). *Basic OECD Principles of GLP. Geneva*.

[21] Parameshappa B., Ali Basha M. S., Sen S. (2012). Acetaminophen-induced nephrotoxicity in rats: protective role of *Cardiospermum halicacabum*. *Pharmaceutical Biology*.

[22] Schwartz G. J., Muñoz A., Schneider M. F. (2009). New equations to estimate GFR in children with CKD. *Journal of the American Society of Nephrology*.

[23] Ellman G. L. (1959). Tissue sulfhydryl groups. *Archives of Biochemistry and Biophysics*.

[24] Hissin P. J., Hilf R. (1976). A fluorometric method for determination of oxidized and reduced glutathione in tissues. *Analytical Biochemistry*.

[25] Reilly C. A., Aust S. D. (2001). Measurement of lipid peroxidation. *Current Protocols in Toxicology*.

[26] L Levine R., Garland D., Oliver C. N., Amici A., Climent I., Lenz A. G. (1990). *Oxygen Radicals in Biological Systems Part B: Oxygen Radicals and Antioxidants*.

[27] Witko-Sarsat V., Friedlander M., Capeillère-Blandin C. (1996). Advanced oxidation protein products as a novel marker of oxidative stress in uremia. *Kidney International*.

[28] Burton K. (1956). A study of the conditions and mechanism of the diphenylamine reaction for the colorimetric estimation of deoxyribonucleic acid. *The Biochemical journal*.

[29] Misra H. P., Fridovich I. (1972). The role of superoxide anion in the autoxidation of epinephrine and a simple assay for superoxide dismutase. *The Journal of Biological Chemistry*.

[30] Aebi H. (1984). Catalase *in vitro*. *Methods in Enzymology*.

[31] Drury R. A. B., Wallington E. A. (1980). *Carleton's Histological Techniques*.

[32] Oyedapo O. O., Akinpelu B. A., Akinwunmi K. F., Adeyinka M. O., Sipeolu F. O. (2010). Red blood cell membrane stabilizing potentials of extracts of *Lantana camara* and its fractions. *International Journal of Plant Physiology and Biochemistry*.

[33] Hossain M., Ahamed S., Dewan S. M. (2014). *In vivo* antipyretic, antiemetic, *in vitro* membrane stabilization, antimicrobial, and cytotoxic activities of different extracts from *Spilanthes paniculata* leaves. *Biological Research*.

[34] Cekmen M., Ilbey Y. O., Ozbek E., Simsek A., Somay A., Ersoz C. (2009). Curcumin prevents oxidative renal damage induced by acetaminophen in rats. *Food and Chemical Toxicology*.

[35] Palani S., Raja S., Kumar R. P., Parameswaran P., Kumar B. S. (2010). Therapeutic efficacy of *Acorus calamus* on acetaminophen induced nephrotoxicity and oxidative stress in male albino rats. *Acta Pharmaceutica Sciencia*.

[36] Jones A. L., Prescott L. F. (1997). Unusual complications of paracetamol poisoning. *Quarterly Journal of Medicine*.

[37] Lorz C., Justo P., Sanz A., Subirá D., Egido J., Ortiz A. (2004). Paracetamol-induced renal tubular injury: a role for ER stress. *Journal of the American Society of Nephrology*.

[38] Adeneye A. A., Benebo A. S. (2008). Protective effect of the aqueous leaf and seed extract of *Phyllanthus amarus* on gentamicin and acetaminophen-induced nephrotoxic rats. *Journal of Ethnopharmacology*.

[39] Yakubu M. T., Bilbis L. S., Lawal M., Akanji M. A. (2003). Evaluation of selected parameters of rat liver and kidney function following repeated administration of yohimbine. *Biochemistry*.

[40] Oloyede O. B., Sunmonu T. O. (2009). Potassium bromate content of selected bread samples in Ilorin, Central Nigeria and its effect on some enzymes of rat liver and kidney. *Food and Chemical Toxicology*.

[41] Shirley D. G., Unwin R. J., Davison A. M., Cameron S. J., Grunfeld J. P. (2005). The structure and function of tubules. *The Oxford Textbook of Clinical Nephrology*.

[42] Devine A., Criddle R. A., Dick I. M., Kerr D. A., Prince R. L. (1999). A longitudinal study of the effect of potassium, sodium and calcium intake on regional bone density in post-menopausal women. *American Journal of Clinical Nutrition*.

[43] Alqasoumi S. I. (2014). Evaluation of the hepatroprotective and nephroprotective activities of *Scrophularia hypericifolia* growing in Saudi Arabia. *Saudi Pharmaceutical Journal*.

[44] Sireeratawong S., Lertprasertsuke N., Srisawat U. (2008). Acute and subchronic toxicity study of the water extract from *Tiliacora triandra* (Colebr.) Diels in rats. *Songklanakarin Journal of Science and Technology*.

[45] Saheed S., Oladipipo A. E., Abdulazeez A. A. (2015). Toxicological evaluations of *Stigma maydis* (corn silk) aqueous extract on hematological and lipid parameters in Wistar rats. *Toxicology Reports*.

[46] Abdul Hamid Z., Siti B. B., Jie N. W., Asmah H., Khairana H., Jamaludin M. (2012). Nephroprotective effects of *Zingiber zerumbet* Smith ethyl acetate extract against paracetamol-induced nephrotoxicity and oxidative stress in rats. *Journal of Zhejiang University of Science (Biotechnology and Biomedicine)*.

[47] Wooley A. (2003). *A Guide to Practical Toxicology Evaluation, Prediction and Risk Determination in General and Reproductive Toxicology*.

[48] Amresh G. R., Singh P. N., Rao C. V. (2008). Toxicological screening of traditional medicine Laghupatha (*Cissampelos pareira*) in experimental animals. *Journal of Ethnopharmacology*.

[49] Das J., Ghosh J., Manna P., Sil P. C. (2010). Taurine protects acetaminophen-induced oxidative damage in mice kidney through APAP urinary excretion and CYP2E1 inactivation. *Toxicology*.

[50] Yousef M. I., Omar S. A. M., El-Guendi M. I., Abdelmegid L. A. (2010). Potential protective effects of quercetin and curcumin on paracetamol-induced histological changes, oxidative stress, impaired liver and kidney functions and haematotoxicity in rat. *Food and Chemical Toxicology*.

[51] Basma K. R., Mona F. S. (2011). The renoprotective effect of honey on paracetamol-induced nephrotoxicity in adult male albino rats. *Life Science Journal*.

[52] Palani S., Raja S., Praveen K. R., Soumya J., Senthil K. B. (2009). Therapeutic efficacy of *Pimpinella tirupatiensis* (Apiaceae) on acetaminophen induced nephrotoxicity and oxidative stress in male albino rats. *International Journal of Pharmacy and Technology Research*.

[53] Kaplowitz N. (2000). Mechanisms of liver cell injury. *Journal of Hepatology*.

[54] Margetis P. I., Antonelou M. H., Petropoulos I. K., Margaritis L. H., Papassideri I. S. (2009). Increased protein carbonylation of red blood cell membrane in diabetic retinopathy. *Experimental and Molecular Pathology*.

[55] Alderman C. J. J., Shah S., Foreman J. C., Chain B. M., Katz D. R. (2002). The role of advanced oxidation protein products in regulation of dendritic cell function. *Free Radical Biology and Medicine*.

[56] Cooke M. S., Evans M. D., Dizdaroglu M., Lunec J. (2003). Oxidative DNA damage: mechanisms, mutation, and disease. *The FASEB Journal*.

[57] Jaeschke H., Bajt M. L. (2006). Intracellular signaling mechanisms of acetaminophen-induced liver cell death. *Toxicological Sciences*.

[58] Brahmi D., Bouaziz C., Ayed Y., Ben-Mansour H., Zourgui L., Bacha H. (2011). Chemopreventive effect of cactus *Opuntia ficus indica* on oxidative stress and genotoxicity of aflatoxin B1. *Nutrition & Metabolism*.

[59] Parlakpinar H., Tasdemir S., Polat A. (2005). Protective role of caffeic acid phenethyl ester (cape) on gentamicin-induced acute renal toxicity in rats. *Toxicology*.

[60] Aslam M., Ahmad S. T., Dayal R. (2012). Nephroprotective action of *Peucedanum grande* against cadmium chloride induced renal toxicity in Wistar rats. *EXCLI Journal*.

[61] Kadir F. A., Kassim N. M., Abdulla M. A., Yehye W. A. (2013). Effect of oral administration of ethanolic extract of *Vitex negundo* on thioacetamide-induced nephrotoxicity in rats. *BMC Complementary and Alternative Medicine*.

[62] Marieb E. (2006). Urinary system. *Essentials of Human Anatomy and Physiology*.

[63] Naggayi M., Mukiibi N., Iliya E. (2015). The protective effects of aqueous extract of *Carica papaya* seeds in paracetamol induced nephrotoxicity in male wistar rats. *African Health Sciences*.

[64] Umukoro S., Ashorobi R. B. (2009). Evaluation of anti-inflammatory and membrane stabilizing property of aqueous leaf extract of *Momordica charantia* in rats. *African Journal of Biomedical Research*.

[65] Amujoyegbe O. O., Agbedahunsi J. M., Akinpelu B. A., Oyedapo O. O. (2012). *In vitro* evaluation of membrane stabilizing activities of leaf and root extracts of *Calliandra portoricensis* (JACQ) benth on sickle and normal human erythrocytes. *International Research Journal of Pharmacy and Pharmacology*.

[66] Khan I., Nisar M., Ebad F. (2009). Anti-inflammatory activities of Sieboldogenin from *Smilax china* Linn.: experimental and computational studies. *Journal of Ethnopharmacology*.

[67] Sabiu S., O'Neill F. H., Ashafa A. O. T. (2016). Kinetics of *α*-amylase and *α*-glucosidase inhibitory potential of *Zea mays* Linnaeus (Poaceae), *Stigma maydis* aqueous extract: an in vitro assessment. *Journal of Ethnopharmacology*.

